# Does Kyphectomy Improve the Quality of Life of Patients With Myelomeningocele?

**DOI:** 10.1097/CORR.0000000000000976

**Published:** 2019-09-19

**Authors:** Pedro Araujo Petersen, Raphael Martus Marcon, Olavo Biraghi Letaif, Marcus Alexandre Mello Santos, Rafael Garcia Oliveira, Tarcísio Eloy Passos de Barros Filho, Alexandre Fogaça Cristante

**Affiliations:** P. A. Petersen, R. M. Marcon, O. B. Letaif, R. G. Oliveira, T. E. Passos de Barros Filho, A. F. Cristante, Spine Group, Faculdade de Medicina, Universidade de São Paulo, São Paulo, Brazil; R. M. Marcon, O. B. Letaif, M. A. M. Santos, A. F. Cristante, Associação de Assistência à Criança Deficiente, Hospital Abreu Sodré, São Paulo, Brazil

## Abstract

**Background:**

Lumbar kyphosis is a complex spinal deformity occurring in approximately 8% to 20% of patients with myelomeningocele. The resulting gibbosity may cause pressure ulcers, difficulty lying down in the supine position and sitting on the ischia without support, decreasing quality of life (QOL). Surgery is generally performed to correct kyphosis and maintain vertebral alignment, but high complication rates have been reported. Despite satisfactory radiological results, the impact of surgery and its complications on health-related QOL (HRQOL) has not yet been established.

**Questions/purposes:**

Among children with myelomeningocele undergoing corrective surgery for lumbar kyphosis: (1) What is the risk of complications and reoperation after this procedure? (2) Does this procedure improve HRQOL scores in these patients?

**Methods:**

Between 2012 and 2013, five surgeons at three centers treated 32 patients for myelomeningocele-related kyphosis with kyphectomy and posterior instrumentation. During that period, all surgeons used the same indications for the procedure, which were progressive postural decompensation and chronic ulceration at the apex of the deformity. Data were prospectively collected, and all patients who underwent surgery were considered in this retrospective study. The legal guardians of one patient declined to sign the informed consent form, resulting in 31 patients included. A total of 9.7% (3 of 31) were lost to follow-up before the 2-year period, and the remaining 90.3% (28 of 31) were seen at a mean of 3 years (± 9 months) after surgery. The average age was 10 years, 7 months (± 21 months) at the time of surgery. The patients had a mean kyphosis angle of 130° ± 36° before surgery. This technique involved posterior fixation using S-shaped rods inserted through the foramina of S1 and pedicle screws inserted in the thoracic spine. The patients’ caregivers answered both the generic and specific (neuromuscular module) Pediatric Quality of Life Inventory questionnaires preoperatively and 2 years postoperatively. The minimum clinically important difference (MCID) considered for the instruments used was 5.

**Results:**

Reoperation was performed in 68% of patients (19 of 28), mostly to treat deep infection. In all, 18% of patients (five of 28) underwent implant removal to control infection. Eleven percent (three of 28) had a loss of reduction and pseudarthrosis. The HRQOL increased from 71 ± 11 preoperatively to 76 ± 10 postoperatively (p < 0.001), resulting in a 5-point increase (95% CI 3 to 7) in the generic questionnaire score and from 71 ± 13 to 79 ± 11 (p < 0.001), resulting in an 8-point increase (95% CI 5 to 10) in the neuromuscular Paediatric Quality of Life Inventory questionnaire score, mainly in the physical health domain on both questionnaires.

**Conclusions:**

Kyphectomy was associated with a high risk of complications and reoperations and did not seem to deliver a substantial clinical benefit for patients who underwent the procedure. Most of our HRQOL score improvements were below the minimum clinically important difference for the Pediatric Quality of Life Inventory questionnaires. Although it seems that surgeons lack a better surgical alternative when facing the challenging health impairments these patients suffer, efforts should be made to improve the technique and reduce surgical complications. Additionally, patients and caregivers should be advised of the high reoperation rate and notified that the procedure may not result in a better QOL and should thus be avoided when possible. Future studies should verify whether decreasing the complication rate could imply improvement in the HRQOL of these patients after surgery.

**Level of Evidence:**

Level IV, therapeutic study.

## Introduction

**Fig. 1 A-B:**
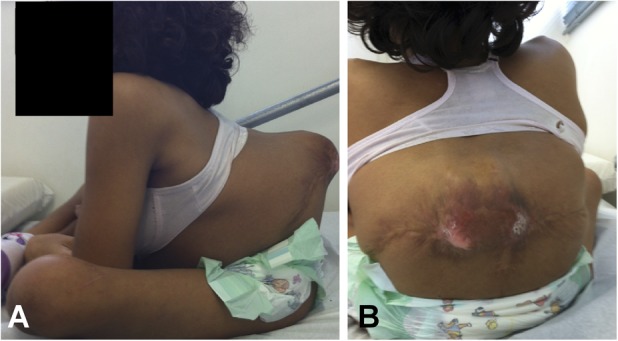
The clinical appearance of a patient with myelomeningocele who shows difficulty in (**A**) sitting without support and (**B**) cutaneous suffering at the apex of the deformity.

Lumbar kyphosis is a complex spinal deformity occurring in approximately 8% to 20% of patients with myelomeningocele [8, 14, 21]. Kyphosis can progress from 6° to 12° per year after birth [2, 3, 6, 24]. Worsening kyphosis is the result of incomplete formation of the posterior spinal elements, an imbalance in the paraspinal musculature anterior to the vertebral axis, unopposed action of the psoas muscle, and neurologic deficits caused by dysraphism [5, 8, 15]. The resulting gibbosity may cause pressure ulcers and bedsores that are difficult to heal, reduce abdominal volume, and result in respiratory compromise. Patients may have difficulty lying down in the supine position and sitting on the ischia because the long lumbar curve includes the sacrum and forces them to support themselves on the posterior sacral lamina (Fig. 1). Surgery is generally indicated to correct kyphosis and maintain vertebral alignment [1, 12, 25].

The surgical correction of kyphosis in patients with myelomeningocele is associated with a high complication rate [9], ranging from 78% to 90% in some series [11, 17, 19]. Most complications appear to be related to the surgical wound, but they also result from instrumentation failure, junctional kyphosis, cerebrospinal fluid fistulae, infections, thromboembolic events, ventriculoperitoneal shunt failure, rhabdomyolysis, meningitis, pneumonia, and urinary tract infections, and can lead to death. Despite the high complication rate, to the best of our knowledge, no prior studies have directly measured the impact of kyphectomy on the quality of life (QOL) of these patients. The objective of this study was to report the health-related QOL (HRQOL) and the results and complications observed in the treatment of kyphosis associated with myelomeningocele in a consecutive series of patients treated using the same surgical technique.

Among children with myelomeningocele undergoing corrective surgery for lumbar kyphosis, we therefore asked: (1) What is the risk of complications and reoperations after this procedure? (2) Does this procedure improve HRQOL scores in these patients?

## Patients and Methods

### Study Design and Setting

This was a retrospective study of a longitudinally maintained database from a series of patients who underwent surgery performed by five surgeons at three university hospitals in São Paulo, Brazil. Patients returned for repeated clinical evaluations. The study protocol was approved by the research ethics committees of the hospitals, and all patients’ legal guardians signed informed consent forms for the procedures and patients’ participation in the study.

### Participants

Between 2012 and 2013, 32 patients with surgical indications attending the three participating institutions were invited to participate in the study. The surgical indications were myelomeningocele with congenital, rigid thoracolumbar kyphosis with progressive postural decompensation leading to difficulty sitting, lying down, and administering care, and chronic ulceration at the apex of the deformity. We excluded those patients who did not consent to have their data used in this study and patients who weighed less than 20 kg. This limit is empirically used by our surgical group to surgically treat pediatric scoliosis or kyphosis, except for growing systems.

### Accounting for All Patients

During the study period, 31 of the 32 patients who underwent surgery at these institutions were included; the legal guardians of one patient declined to sign the informed consent form to participate in the study. Subsequently, three patients who did not complete the minimum 2-year follow-up period were excluded, resulting in 28 analyzed patients.

### Demographics, Description of Study Population

The mean follow-up time for the patients who completed the study was 3 years (± 9 months). Eight of 28 patients (29%) were male. The average age at the time of surgery was 10 years, 7 months (± 20 months), and the mean weight was 30 kg (± 10 kg). Sixty-one percent of patients (17 of 28) had test results that were positive for latex sensitivity.

### Surgical Technique and Perioperative Care

Patients with a ventriculoperitoneal shunt only underwent surgery if the shunt was permeable and their hydrocephalus was compensated for surgery. Urine cultures were used to guide the treatment of possible infections with at least 3 days of antibiogram-guided antibiotic therapy before surgery. Antibiotic prophylaxis was administered in the form of first-generation cephalosporin. All patients were tested for a latex allergy, which is common in patients with myelomeningocele [13, 18]. In patients whose test result was positive, a 100% latex-free environment was prepared in the surgical center.

The surgical technique used was a modified Dunn-McCarthy technique [16]; fixation of the thoracic spine was performed using pedicle screws instead of sublaminar wires. Screws (USS 1 titanium pedicle screws [DePuy Synthes Spine, Raynham, MA, USA]) were inserted proximally into the normal thoracic spine. The number of fixation points for the interface between the screw and bone was determined subjectively by the surgeon. Six screws in three vertebrae were frequently used.

**Fig. 2 A-C:**
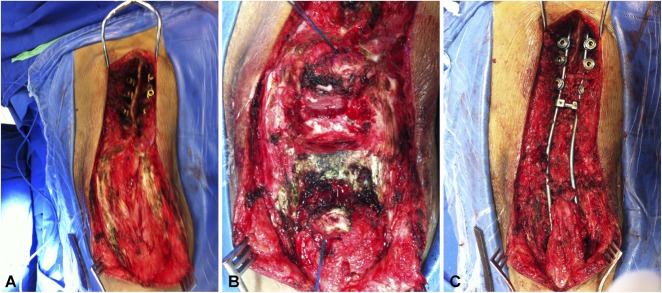
Intraoperative images demonstrating the surgical technique. (**A**) Dissection and insertion of the proximal screws, (**B**) exposure of the apex of kyphosis and cordotomy, and (**C**) kyphectomy and reduction of the deformity with rod fixation in the foramina of S1 are shown.

At the apex of the kyphosis, dissection was performed and restricted to the region of the transverse processes because there was no lamina protecting the dural sac in this area (Fig. 2A). Sometimes there was no dural sac at the apex of the deformity. This may occur because of the underlying cause, for example, fibrosis caused by pressure ulcers. The thecal sac was then sutured with 4/0 Prolene® (J&J Medical Devices, New Brunswick, NJ, USA), and cordotomy was performed above the level of the planned osteotomy (Fig. 2B). Next, vertebrectomy of the apex of the kyphosis was performed, and the vertebrae were removed as necessary to achieve good apposition of the preserved vertebral bodies. An attempt was made to preserve at least L4 and L5 so that in addition to the sacrum, there was a sufficient area of bone support and arthrodesis.

Osteosynthesis was then performed following the principles of the Dunn-McCarthy technique [16]. The rods were bent into the shape of a bayonet (S-shaped), and the short arms of the rods were passed through the foramina of S1 so they were anterior to the sacrum [29]. By orienting the rods horizontally, a lever was created that reduced lumbar kyphosis and restored the sacrum to its original position. The upper portions of the rods were then fixed to the thoracic spine using pedicle screws. The resected body provides a good amount of bone graft material (Fig. 2C). Postoperative immobilization was not used.

### Variables and Data Collection

Before the surgical procedures, demographic and anthropometric data were collected, and spinal kyphosis was measured using the Cobb method. The surgical information included the operative time, number of red blood cell transfusions, proximal level of the arthrodesis, number of vertebrae removed, postoperative angle reduction, and hospitalization duration after the procedure. Complications related to postoperative infections, cerebrospinal fluid fistulae, acute hydrocephalus, loosening or breakage of the synthesis material, and pseudarthrosis were also recorded.

**Fig. 3 A-C:**
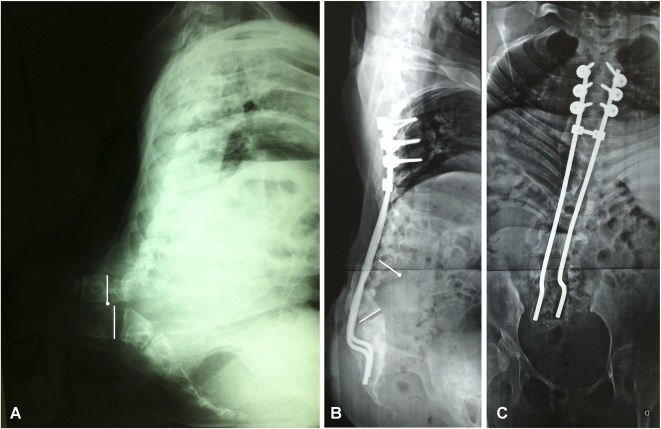
A preoperative radiograph of a patient showing (**A**) lumbar kyphosis of 175°. (**B**-**C**) Postoperative radiographs showing correction of kyphosis of 65°.

The average duration of surgery was 246 ± 71 minutes from the first incision to the last suture. An average (range) of two vertebral bodies were removed (one to four). The patients received an average (range) of 0.6 units of red blood cells during the procedure (0 to 2). The mean (range) hospitalization duration was 14 days (4 to 45). The average preoperative kyphosis angle of the patients was 130° ± 36°, which was corrected to 56° ± 29° during the immediate postoperative period, representing an average correction of 58% (Fig. 3). After 2 years, the mean kyphosis angle of the patients was 60° ± 30°, representing a correction of 53%.

### Follow-up Routine

The patients returned for postoperative follow-up evaluations at 2 weeks, 6 weeks, 12 weeks, 6 months, 12 months, and 24 months postoperatively. Other follow-up visits were conducted according to the specific needs of each patient.

### Outcome Measures and QOL Evaluation

The patients underwent a QOL analysis preoperatively and 2 years postoperatively using two questionnaires: the Pediatric Quality of Life Inventory (PedsQL) 4.0 generic core scale, which is a general questionnaire, and the PedsQL 3.0 neuromuscular module, which is a condition-specific questionnaire. The questionnaires were completed by the patients’ parents or legal guardians. Both questionnaires have been translated into Portuguese and culturally adapted for the Brazilian population [26-28].

The MCID considered for the generic core scale was 5, as established previously in the evidence for the generic core scale using distribution-based methods (that is, standard error of measurement) [7, 28]. We found no previous studies establishing the MCID for the neuromuscular module.

### Statistical Analysis

For the data analysis, descriptive measurements of the patients’ characteristics, such as age, sex, and preoperative Cobb angle, were recorded in spreadsheets and are reported as frequencies, means, and SDs, as were data regarding the operative time, number of vertebrae involved, and number of blood transfusions. Postoperatively, the number of days in the hospital and types of complications were recorded for each patient.

For the inferential analysis, the Shapiro-Wilk test for data distribution normality was performed on all continuous data, in addition to a subjective histogram analysis. The Cobb angle at the preoperative, immediate postoperative, and 2-year postoperative time points were compared using repeated-measures ANOVA. The Wilcoxon signed-rank test was performed to analyze the preoperative and postoperative QOL, and this test was used separately in patients with complications and those without complications. Association and correlation analyses of the QOL variables and the presence of complications were performed using the Pearson chi-square test or Fisher’s test.

The PedsQL subscale scores were calculated using the algorithm provided by the creators of the questionnaire [27]. The results range from 0 to 100, with higher scores indicating a better HRQOL. If more than 50% of the items on a subscale were missing, the result was not recorded. For inferential statistics, a test for paired samples—a t-test or the Wilcoxon signed-rank test—was used.

The level of statistical significance (probability of a type I error) used in this study was 0.01. The software used for the statistical analysis was SPSS 23.0 for Macintosh (IBM Corp, Armonk, NY, USA).

## Results

Complications after this procedure were common, as were reoperations. In all, 68% of patients (19 of 28) underwent reoperation. Sixty-four percent of patients (18 of 28) underwent débridement to treat deep infection, and one patient experienced rod fracture after an automobile collision. One patient developed a cerebrospinal fluid fistula, and surgery and suturing were performed a second time. Fourteen percent of patients (four of 28) underwent plastic surgery with an advancement flap for cutaneous coverage. Eighteen percent of patients (five of 28) underwent implant removal to control infection. There was a loss of reduction and pseudarthrosis in 11% (three of 28). No patients had acute hydrocephalus or anaphylaxis resulting from latex allergies.

The HRQOL improved slightly on both questionnaires used but did not exceed the MCID. The generic core scale questionnaire score increased from 71 ± 11 preoperatively to 76 ± 10 postoperatively (p < 0.001), resulting in a 5-point increase (95% CI 3 to 7) after 2 years (Table 1). This improvement was mainly seen in the physical health component. There were no differences when the emotional, social, and educational aspects of the generic core scale were evaluated separately. The results of the neuromuscular module showed an improvement from 71 ± 13 to 79 ± 11 (p < 0.001), resulting in an 8-point increase (95% CI 5 to 10) after 2 years, also mainly in the physical component of the scale (Table 2). Once again, there were no differences in the other aspects of the scale (family and communication) when these items were analyzed separately. There was no difference in HRQOL improvement on either scale between patients who underwent surgical reintervention and those who did not. However, when analyzed separately, there was no improvement in the generic core scale-based or neuromuscular module-based QOL of the three patients who showed a loss of reduction compared with the rest of the patients.

**Table 1.**
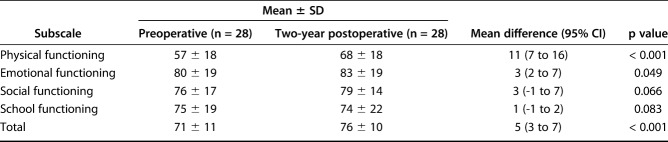
Health-related quality of life scores for the generic core scale questionnaire

**Table 2.**
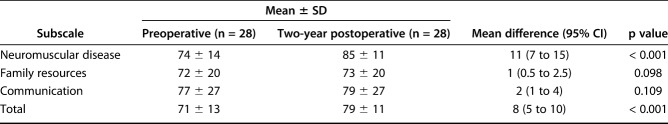
Health-related quality of life scores for the neuromuscular module of the questionnaire

## Discussion

Kyphectomy in patients with myelomeningocele has historically been performed with a high associated complication rate [9, 11, 17, 19]. Most complications appear to be related to surgical site infection but also occur due to implant failure and pseudarthrosis. Despite the high complication rate, previous case series did not seek to directly measure the impact of kyphectomy on the QOL of these patients. Our series confirmed a high reoperation rate of 68%, with an 11% loss of reduction and pseudarthrosis. HRQOL improved slightly but did not exceed the MCID. Surgical treatment appears to offer low clinical benefit for these patients as it is associated with a high morbidity rate.

One limitation of our study is that because it involved a series of patients with a small sample size (despite being one of the largest series published so far), there was no control group. An effort was made to use the same indications, technique, and standardized measurement tools to render our results comparable to those of future studies. Further improvement of the technique as suggested by us or others could yield better and comparable results. Additionally, because this study was conducted in academic hospitals that are national references, patients could potentially have a worse prior condition and thus have worse surgical results and be more susceptible to complications. Also, 9.7% of our patients were lost to follow-up. This transfer bias could imply that these patients could have a worse outcome than the ones who completed the minimum follow-up period. Although we have found references to the generic questionnaire MCID in the evidence [4, 28], we found none regarding the MCID for the neuromuscular module. Despite this fact, it is unlikely that it would be lower than 5 on a 100-point scale, especially considering that the clinically perceptible change tends to be higher for surgical interventions. Although both the generic and neuromuscular modules have been validated in independent studies [4, 28], only the generic core scale has been validated for Portuguese speakers [10]. A lack of congruence between a child and a proxy (usually a parent) has been reported, especially in the emotional and social domains [23]. This could imply different perceptions of change if the questionnaire was answered only from the patient’s point of view, even if this version of the questionnaire has been proven reliable [28]. The neuromuscular module, although translated, has not yet been used in the Portuguese language in an independent study. Despite this fact, there are a limited number of measurement tools applicable in Portuguese, so we chose this questionnaire because it has been widely used in the English language and was properly translated.

### Complications and Reoperations Following Kyphectomy

**Fig. 4 A-C:**
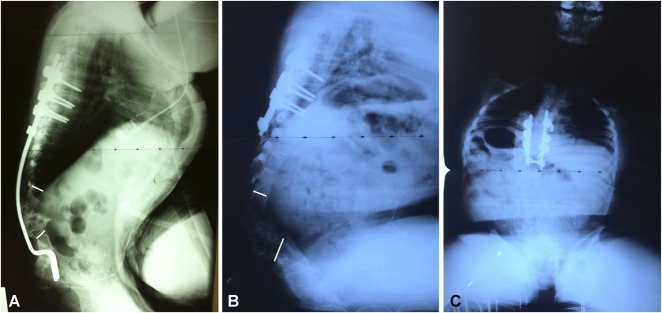
Postoperative radiographs showing (**A**) immediate postoperative correction of 62°. (**B**-**C**) After infection and implant removal, 2-year postoperative radiographs showing a loss of reduction and a Cobb angle of 98°.

The thin, poor-quality skin used to cover the surgical area may influence the postoperative infection rate. However, implant removal did not necessarily imply a loss of reduction and pseudarthrosis during the long-term follow-up period. This is consistent with the findings of Altiok et al*.* [1], which showed that seven of 21 patients had implant failure but only three required revision. Implant removal does not always lead to loss of reduction, as three of five patients who had implants removed in our series showed solid fusion and maintained the correction gained. Although high rates of nonunion have been reported (such as 15% and 40%) [1, 29], pseudarthrosis does not necessarily lead to revision surgery (Fig. 4). The absence of pain and the removal of vertebral bodies with consequent gibbous reduction can lead to the resolution of wound-related complications. Nevertheless, when we analyzed them separately, of the three patients who underwent reoperation for an infection and developed a loss of reduction, none had an improved QOL after 2 years. This subgroup was too small for us to statistically infer that a loss of reduction had a direct impact on QOL, although clinical observations suggested this phenomenon. A larger sample size could potentially clarify this matter. Rod loosening in the sacrum is a complication of this technique, which uses only the foramina of S1 as a distal anchoring point. One way to improve the technique would be to use more anchoring points, such as iliac screws. Another possibility [9] is to use fixation with pedicle screws in the segments with dysraphism or a technique using S1 screws with S2 alar-iliac screws [6]. It remains to be seen whether these alternative techniques can reduce the incidence of complications related to the distal loosening of implants. One study recommended surgery without myelotomy, reporting that it reduces the chances of complications related to increased intracranial pressure, the formation of a cerebrospinal fluid fistula, and meningitis [20]. Only one of our patients developed a cerebrospinal fluid fistula, and no complications related to ligation of the dural sac or failure of the ventriculoperitoneal shunt (acute hydrocephalus) were observed.

### HRQOL Scores Following Kyphectomy for Myelomeningocele

In addition to the frequent, severe complications we have already discussed, surgery did not seem to improve the patients’ HRQOL scores by a clinically important amount. The MCID of 5 stayed inside the 95% confidence interval for the generic and neuromuscular scales. Although condition-specific questionnaires tend to capture some aspects that are not covered by generic questionnaires because they target a subgroup of patients, even the more specific neuromuscular questionnaire showed an improvement very close to the MCID (Table 2). The MCID for the generic core scale was reported to be 4.5 or 5.16 when calculated based on the standard error of measurement [7, 28]. It was not determined in surgical patients, but it was determined in patients undergoing less extensive health interventions. Therefore, it is possible that the MCID would be higher if calculated in a surgical population using an anchor-based approach. Previously published series have reported high levels of satisfaction with surgery (nine of 11 patients) [22], but it seems that these findings did not translate into perceptible changes in HRQOL.

### Conclusions

Kyphectomy is associated with a high risk of complications and reoperations and did not seem to deliver a substantial clinical benefit for patients who underwent the procedure. Most of our HRQOL score improvements were below the minimum clinically important difference for the Pediatric Quality of Life Inventory questionnaires. Although it seems that surgeons lack a better surgical alternative when facing the challenging health impairments these patients suffer, efforts should be made to improve the technique and reduce surgical complications. Additionally, patients and caregivers should be advised of the high reoperation rate and notified that the procedure may not result in a better QOL and should thus be avoided when possible. Future studies should verify whether decreasing the complication rate could imply improvement in the HRQOL of these patients after surgery.
